# Shifting Career Landscapes: Analyzing the Fall in Physician-Owned Practices

**DOI:** 10.26502/acmcr.96550750

**Published:** 2026-03-17

**Authors:** Andre Aabedi, Vera Wang, Ramtin Sahafi, Devendra K Agrawal

**Affiliations:** Department of Translational Research, College of Osteopathic Medicine of the Pacific, Western University of Health Sciences, Pomona, California 91766 USA

**Keywords:** Administrative burden, Healthcare consolidation, Healthcare costs, Physician autonomy, Physician employment, Private practice

## Abstract

Over the past several decades, the structure of physician employment in the United States has shifted markedly, with a growing proportion of physicians choosing employed positions over private practice. This transition reflects broader economic, regulatory, and organizational changes within the healthcare system. This literature review examines the decline in physicians entering private practice and analyzes the economic, administrative, professional, and market-driven factors contributing to this trend, as well as its implications for physician autonomy, patient care, and healthcare costs. A comprehensive review of national workforce data, longitudinal studies, and peer-reviewed literature published between 1983 and 2024 was conducted. Trends in physician employment were evaluated across specialties, geographic regions, and demographic groups, with particular attention to policy changes, healthcare consolidation, and evolving practice models. The proportion of self-employed physicians declined from 35.2% in the early 2000s to under 25% by the mid-2010s, with continued declines in subsequent years. Contributing factors include rising overhead costs, declining reimbursement, increasing regulatory and administrative burdens, and narrowing income differentials between employed and self-employed physicians. Younger physicians and women demonstrate a stronger preference for employment, often citing work-life balance, financial stability, and organizational support. Healthcare consolidation, hospital employment, and private equity acquisition further limit opportunities for independent practice. The ongoing decline of private practice has significant implications for physician autonomy, continuity of care, healthcare spending, and patient-physician relationships. While employed models offer stability and infrastructure, they may compromise professional independence and increase system-level costs without improving care quality. Alternative models such as direct primary care and concierge medicine may offer partial solutions but raise concerns regarding equity and access. Structural reforms in reimbursement, regulation, and practice support are necessary to preserve a diverse and sustainable physician workforce.

## Introduction

The landscape of physician employment has undergone significant changes over the past few decades, with a notable decline in the percentage of physicians entering private practice. Several factors have contributed to this shift, including economic pressures, changes in healthcare delivery models, and evolving physician preferences. From 2001 to 2015, the percentage of self-employed physicians decreased from 35.2% to 24.7% [1]. This trend is consistent across various specialties, including general surgery, where the proportion of self-employed surgeons dropped from 48% to 33% between 2001 and 2009 [2]. The American Medical Association’s Socioeconomic Monitoring System also reported a rise in the proportion of employed physicians from 24.2% in 1983 to 42.3% in 1994 [3]. The growth of Accountable Care Organizations (ACOs) has been associated with a reduction in self-employment among physicians. A study found that a 10-percentage point increase in ACO enrollment was linked to a 2% decrease in the probability of physicians being self-employed [4]. Additionally, the shift towards employment is more pronounced in rural areas, where the decline in independent solo practices has been significant [5]. Economic factors also play a role. The earnings gap between self-employed and employed physicians has narrowed, with employed physicians sometimes earning more than their self-employed counterparts [1]. This economic incentive, combined with the administrative burdens of running a private practice, has made employment more attractive. Private practice has historically been a cornerstone of healthcare delivery, offering physicians autonomy, flexibility, and the ability to build long-term patient relationships. However, the landscape is shifting, with a notable decline in the percentage of physicians entering private practice. Several factors contribute to this trend. Economic pressures, such as the rising costs of maintaining private practice and the financial stability offered by employment, play a significant role. According to a study published in JAMA Network Open, the percentage of self-employed physicians decreased from 35.2% in 2001–2005 to 24.7% in 2011–2015, with a corresponding narrowing of the earnings gap between self-employed and employed physicians [1]. This shift is also observed in other healthcare professionals, indicating a broader trend within the sector. Additionally, regulatory and administrative burdens have increased, making private practice less attractive. The American College of Physicians highlights that the growing emphasis on the business aspects of medicine and regulatory requirements can impact the ethical and professional responsibilities of physicians [6]. This environment can deter new physicians from pursuing private practice, favoring employment in larger organizations that can absorb these administrative tasks. Rural areas are particularly affected by these changes. A longitudinal study in South Carolina found a significant shift towards physician employment across all levels of rurality, with rural counties experiencing a more pronounced transition [5]. This shift can impact access to care in underserved areas, where independent practices have traditionally played a crucial role. The purpose of this literature review is to assess the declining percentage in the percentage of physicians going into private practice.

## Historical Perspective on Physician Private Practice

The evolution of private practice in the United States has undergone significant changes over the past century. Historically, physicians predominantly operated in small, independently owned practices, often as solo practitioners. This model was prevalent until the mid-20th century, when the landscape began to shift due to various socioeconomic factors. In the latter half of the 20th century, there was a notable trend towards physicians becoming employees rather than owners of their practices. Between 1983 and 1994, the proportion of patient care physicians practicing as employees increased from 24.2% to 42.3%, while those in solo practices decreased from 40.5% to 29.3% [3]. This shift was driven by changes in the healthcare system, including the rise of managed care, increased regulatory requirements, and the financial pressures of maintaining private practice. By the early 21st century, the trend towards employment continued, with a significant number of surgeons and other specialists opting for hospital or large group employment. Between 2001 and 2009, the number of self-employed surgeons decreased from 48% to 33%, with a corresponding increase in employed surgeons [2]. This shift was particularly pronounced among younger and female physicians, who favored the stability and resources provided by larger organizations. The American College of Physicians has highlighted the ethical and professional implications of this trend, noting that the increasing employment of physicians and changing practice models can impact the patient-physician relationship and the primacy of patient welfare [6]. Additionally, the rapid movement from small to large group practices between 2013 and 2015 further underscores the consolidation trend in the physician workforce [7].

Historically, several traditional incentives have driven physicians to enter private practice. One of the primary incentives has been professional autonomy. Private practice allows physicians to have greater control over their clinical decisions, work schedules, and practice management, which can be highly appealing compared to the constraints often found in employed positions [8]. Financial incentives have also played a significant role. Private practice historically offered the potential for higher earnings through fee-for-service models, where physicians could directly benefit from the volume and type of services provided. This financial independence was a strong motivator for many physicians [9]. Patient relationships and continuity of care are other important factors. In private practice, physicians often can build long-term relationships with their patients, providing a sense of personal and professional fulfillment that can be less prevalent in larger, more impersonal healthcare systems [8]. Additionally, entrepreneurial opportunities have been a draw [10]. Physicians in private practice can innovate and adapt their practice to meet patient needs and market demands, fostering a sense of ownership and investment in their work [11]. Lastly, geographic flexibility has been a traditional incentive. Physicians in private practice can choose their practice location, allowing them to serve specific communities or underserved areas, aligning with personal or professional goals [12]. These incentives have historically made private practice an attractive option for many physicians, although recent trends indicate a shift towards employment models due to changing economic and regulatory landscapes [13]. Private practice has played a significant role in patient care and physician autonomy. Private practice allowed physicians to exercise considerable control over their clinical decisions, patient interactions, and business operations. This autonomy was seen as a cornerstone of medical professionalism, enabling physicians to prioritize patient welfare without external interference [3]. However, the landscape of physician employment has shifted dramatically over the past few decades. The proportion of physicians in private practice has declined, with more physicians becoming employees of larger healthcare organizations. This trend is driven by various factors, including economic pressures, regulatory changes, and the increasing complexity of healthcare delivery [14]. The American College of Physicians has highlighted the ethical and professional implications of this shift, noting that while employment models can offer stability and resources, they may also introduce conflicts between business interests and patient care priorities. Additionally, research indicates that while employed physicians may experience reduced autonomy in logistic-based decisions, they may still retain significant control over clinical decisions, suggesting a nuanced impact on professional autonomy [15].

## Current Trends in Physician Employment

The statistical analysis of the shift in physician employment from private practice to employed settings reveals significant trends over recent decades. Between 1983 and 1994, the proportion of patient care physicians practicing as employees increased from 24.2% to 42.3%, while those in solo practices decreased from 40.5% to 29.3%, and those in group practices fell from 35.3% to 28.4% [3]. This trend is particularly pronounced among younger physicians, suggesting a generational shift in practice preferences. A longitudinal study in South Carolina from 1995 to 2015 showed a continual increase in physician employment, with an average annual increase of 5.9%. Micropolitan rural counties exhibited the highest average annual increase in employed physicians at 7.4% [5]. This indicates that the shift towards employment is not confined to urban areas but is also significant in rural settings. For surgeons, the trend is similar. From 2001 to 2009, the number of self-employed surgeons decreased from 48% to 33%, with a corresponding increase in employed surgeons. By 2011, 68% of surgeons identified as employed, with younger and female surgeons showing a stronger preference for employment in large group practices [2]. The shift in physician employment from private practice to hospital and corporate employment models has been driven by several factors, including regulatory changes, economic pressures, and evolving healthcare delivery models. The Medicare Access and CHIP Reauthorization Act of 2015 (MACRA) has played a significant role in accelerating the movement of physicians into corporate employment. MACRA’s emphasis on value-based care and its complex reporting requirements have made it challenging for small independent practices to thrive, pushing many physicians towards employment by larger entities that can better manage these demands [16]. A study by Charles and co-investigators [2] highlights that the number of surgeons in self-employed practices decreased significantly from 48% to 33% between 2001 and 2009, with a corresponding increase in hospital employment. This trend is particularly pronounced among younger and female surgeons, indicating a shift in professional paradigms [2]. Kletke et al. observed a similar trend across all specialties, noting that the proportion of patient care physicians practicing as employees rose from 24.2% to 42.3% between 1983 and 1994. This shift is associated with increased earnings for employee physicians relative to their self-employed counterparts [3].

Scott and colleagues [17] found that the proportion of hospitals employing physicians increased from 29% in 2003 to 42% in 2012. However, this shift did not necessarily translate into improved quality of care, suggesting that physician employment alone is not sufficient to enhance hospital performance [17]. The American College of Physicians has also discussed the ethical and professional implications of this shift, emphasizing the need for physicians to maintain their commitment to patient welfare despite the changing practice environment [6]. The declining percentage of physicians entering private practice is influenced by several factors, including economic, professional, and personal considerations. Economics factors play a significant role. The shift towards employment models is driven by the financial stability and benefits offered by hospital systems and large group practices. Employed physicians often receive higher relative earnings compared to self-employed solo practitioners, as noted in the study by Kletke et al [3]. Additionally, the increasing complexity and cost of running a private practice, including administrative burdens and regulatory requirements, make employment more attractive. Professional factors also contribute to this trend. Younger physicians and female physicians are increasingly favoring employment in large group practices due to the structured environment and support systems available [18]. Employment models can offer better work-life balance, reduced administrative responsibilities, and more predictable schedules, which are appealing to many physicians. Personal considerations, such as job satisfaction and burnout, are critical. Physicians report higher satisfaction with leisure and family time in employed settings [19]. The stress associated with electronic health records (EHRs), compensation models, and leadership support are significant factors influencing physicians’ decisions to leave private practice for employed positions [20]. Ethical and professionalism implications are also relevant. The American College of Physicians highlights the need for physicians to consider how employment models affect their ethical and professional responsibilities, including the primacy of patient welfare and the integrity of the patient-physician relationship [21].

## Factors Contributing to the Decline of Private Practice

Rising overhead costs and declining reimbursement rates are major contributory factors leading to the decline of physicians entering private practice. Maintenance of a viable private practice entails significant investment in administrative and healthcare staff, electronic health records, malpractice insurance, medical infrastructure and equipment and their respective maintenance fees, all of which have climbed in price over time. Additionally, reimbursement rates from Medicare, Medicaid, and private insurers have not reached equilibrium with these costs and often fail to cover the full scope of cost of healthcare delivery. These financial hurdles make private practice increasingly unviable, especially for newly entering physicians with significant medical school debt. Therefore, there is an increasing preference to be a part of a larger healthcare system that offers financial stability, back-end support, and less medical liability. A 2022 report by the American Medical Association states that only 46% of physicians now own their practices, down from 60% back in 2012, which continues a stable trend away from private practice due to such financial obstacles [22]. Additionally, maintaining profitability is yet another challenge in private practice. Private practitioners must essentially operate a dual-pronged approach, both as a healthcare provider and a business owner, guiding themselves through a complex and shifting financial landscape, as well as preparing for ebbs and flows within the macro-economy. Obstacles such as increasing administrative burdens, rising labor and supply costs, and reduced negotiating power with insurers make it difficult to sustain a profitable practice without injection of capital to tide over razor-thin margins. This ties into the benefits of joining a larger healthcare system, as physicians often lack the substantial resources and economies of scale that larger healthcare systems benefit from, putting these physicians at a competitive and financial disadvantage. Such financial pressures not only threaten long-term viability of current private practices but also discourage new physicians from starting new practices. A report by The Journal of the American Board of Family Medicine found that the proportion of primary care physicians practicing in organizations owned by a health system or hospital increased from 28% in 2010 to 44% in 2016 [23].

Physicians in private practice now face intensified regulatory and administrative challenges due to expanded compliance obligations and increasingly complex insurance and billing systems. Healthcare professionals need constant vigilance and often hire either specialized staff or expensive consultants to manage ongoing updates to HIPAA and policies for Medicare and Medicaid. Handling various insurance providers and differing reimbursement methods along with obtaining necessary preauthorization’s requires extensive time commitment and resources which shift focus away from direct patient treatment. The increasing demands of regulatory compliance and financial management responsibilities drive physician burnout while making independent practice unsustainable. Billing challenges are also an immense burden with nearly 11 to 54 billion dollars in challenged revenue annually due to billing complexities. According to Health Affairs research, physicians dedicate almost two hours to administrative work for each hour they spend on clinical tasks because billing and insurance activities consume most of their time [24].

The effects of market consolidation and corporate influence, including private equity (PE) and hospital acquisitions, on physician autonomy and patient care are multifaceted. PE involvement has been rapidly growing and often prioritizes financial gains, which can lead to increased physician turnover, reduced autonomy, and potential negative impacts on patient care quality. This is exacerbated by the desire for quick returns, which PE firms typically look for within 3–5 years [25]. A systematic review by Borsa et al. found that PE acquisitions have been formatted with increased costs for patients and mixed harmful impacts on care quality [26]. Critics highlight that such business practices can erode the patient-physician relationship and place profit motives above patient welfare [16,27]. Physician work-life balance and lifestyle preferences, such as predictable hours and reduced administrative work, significantly impact burnout and stress levels in private practice. Studies indicate that physicians in corporate-owned practices may experience lower work-life balance and higher burnout rates due to increased administrative burdens and reduced autonomy [28,29]. Conversely, opportunities for internal recovery and a balance between work and private life are associated with lower emotional exhaustion and burnout [30]. Therefore, addressing these factors is crucial for maintaining physician well-being and ensuring high-quality patient care.

## Implications for Healthcare and Patient Outcomes

The decreasing number of doctors choosing private practice affects multiple aspects of healthcare including service costs, patient access, doctor-patient interactions, and overall care quality. The employment of physicians by larger healthcare systems gives these organizations stronger bargaining power with insurance companies which pushes service prices up and increases healthcare costs for patients. This consolidation process may diminish competition which subsequently restricts patients from accessing a variety of healthcare providers and services. The movement of doctors from private practices into larger employment system changes how doctors interact with patients. Physicians working in private practice settings usually have the freedom to establish lasting personal connections with their patients which helps build trust and ensure continuous care. Physicians who work within organizations face structural limitations that prevent them from building effective doctor-patient relationships which can reduce both patient satisfaction and treatment results. Additionally, financial bridging between physicians and hospitals raises patient spending without increasing care quality, with data illustrating that patients who received treatment from hospital-owned practices faced 5.8% higher annual costs than those treated by physician-owned practices [31]. The quality of care remained similar across both settings even though the costs were higher. The data indicates that when doctors and hospitals merge financially, they can increase healthcare spending on patients without improving the quality of care. For instance, rather than rewarding the acquisition and integration of practice characteristics such as a larger systems-based approach and non-physician providers, it might be better simply to reward physicians directly for better screening and monitoring, lower levels of avoidable utilization, and lower risk-adjusted cost [32]. The transition of physicians from private practice to employment in bigger healthcare systems has major consequences for healthcare expenses and access alongside its impact on both the doctor-patient connection and the quality of medical services.

## Future Directions and Potential Solutions

Increasing primary care spending and adjusting reimbursement rates for inflation is essential. This can be achieved through state-level legislation, increasing Medicare’s reimbursement towards primary care and efforts by health systems to allocate more resources to primary care. Shifting from productivity-based compensation to models that value quality of care and physician well-being can additionally alleviate the pressure to see a high volume of patients, thus allowing for more predictable hours and better work-life balance. Alternative business models can help physicians adjust to the changing landscape of medicine. Concierge medicine is a popular growing model and involves patients paying a retainer fee for more personalized and accessible care. While it can increase patient and physician satisfaction, it raises ethical concerns about access for underserved populations [33–34]. Direct care models, where patients pay a monthly or annual fee directly to the practice, can reduce administrative burdens associated with insurance billing and provide more predictable revenue streams. Financial analysts suggest DPC can be cost-effective and transformative for the healthcare landscape [34]. New technologies have helped expand healthcare access and reduce administrative burdens through telehealth and advanced health information technology systems. Telehealth allows for more flexible and efficient patient management, with the option to work from home, however its future regarding reimbursements remains unclear [35]. Artificial intelligence has shown promising results for decreasing documentation time and even performing tasks typically assigned to a medical assistant, however this may only reduce physician burnout if it is not countered by increasing patient load [36].

## Conclusions

There is a consistent and significant decline in the percentage of physicians entering or remaining in private practice, driven by a range of economic, administrative, and professional factors. Financially, the rising costs of maintaining a private practice, including staffing, electronic health records, malpractice insurance, and equipment, combined with declining reimbursement rates, have made self-employment increasingly difficult especially for newer physicians. Regulatory and administrative complexities, including compliance with Medicare, Medicaid, and insurance billing systems, further diverting time and resources away from patient care and contribute to high levels of burnout. These challenges have coincided with a generational and gender shift in practice preferences, with younger and female physicians favoring employment in larger organizations due to the perceived benefits of work-life balance, predictable schedules, and organizational support. Healthcare consolidation through hospital acquisitions and the growing influence of private equity has also played a major role, further shifting the practice landscape. These trends have implications for both physician autonomy and patient care, with concerns that financial incentives and corporate priorities may undermine the patient-physician relationship and reduce continuity of care. Although employment models may offer economic stability and infrastructure, they are often associated with reduced clinical independence and strained relationships due to time constraints and institutional priorities. Despite these shifts, alternative practice models such as concierge medicine and direct primary care are emerging as viable options, offering reduced administrative burdens and more personalized care. However, these models raise questions about equitable access, particularly for underserved populations. While existing literature provides a robust understanding of the economic and policy-driven factors contributing to the decline of private practice, several gaps remain. There is limited qualitative research capturing the lived experiences and nuanced decision-making processes of physicians transitioning out of or avoiding private practice. Additionally, more longitudinal data are needed to understand how these shifts affect patient outcomes over time, particularly in rural and underserved communities where private practices have historically played a vital role. Future research should focus on evaluating the long-term effects of market consolidation on healthcare quality and access, the efficacy and scalability of alternative business models, and the role of technology in sustaining independent practices. Artificial intelligence is an especially new tool that has a promising role in decreasing burnout and reducing documentation, however, needs more large-scale studies and affordability. Policymakers and healthcare leaders must consider structural changes that support private practice viability, including payment reform, regulatory simplification, and investment in primary care infrastructure.

## Key points

- The proportion of physicians entering or remaining in private practice has steadily declined over the past four decades.
- Rising overhead costs and stagnant or declining reimbursement have made private practice increasingly financially unsustainable.
- Administrative and regulatory burdens divert physician time away from patient care and contribute significantly to burnout.
- Younger and female physicians are more likely to prefer employed positions due to work-life balance and income stability.
- Hospital systems and private equity firms have accelerated consolidation, reducing independent practice opportunities.
- Employed practice models often increase healthcare costs without demonstrable improvements in care quality.
- Private practice historically supported physician autonomy, continuity of care, and long-term patient relationships.
- Alternative models such as direct primary care and concierge medicine reduce administrative burden but raise equity concerns.
- Policy reform and payment restructuring are essential to preserve independent practice and maintain workforce diversity.
